# Physicochemical and Sensory Properties of Pork Patties with Partial Replacement of Lean Pork by Stalks of *Agaricus bisporus*

**DOI:** 10.3390/foods14213655

**Published:** 2025-10-27

**Authors:** Liyan Wang, Shuo Li, Huajie Tu, Xiaoxia Yan, Zhiqing Hu, Hongrui Sun

**Affiliations:** 1 College of Food Science and Engineering, Jilin Agricultural University, 2888 Xincheng Street, Changchun 130118, China; wangliyan@jlau.edu.cn (L.W.); shuoli20241088@163.com (S.L.); 20200816@mails.jlau.edu.cn (H.T.); 2 College of Food Science and Engineering, Jilin University, 5333 Xi’an Road, Changchun 130062, China; xxyan@jlu.edu.cn; 3 College of Engineering and Technology, Jilin Agricultural University, 2888 Xincheng Street, Changchun 130118, China; 4 Institute of Agro-Food Technology, Jilin Academy of Agricultural Sciences, 1363 Shengtai Street, Changchun 130033, China; hongruisun@163.com

**Keywords:** *Agaricus bisporus*, mushroom, pork patties, meat substitute

## Abstract

Pork patties were prepared by replacing lean pork with *Agaricus bisporus* (AB) at 25%, 50%, 75%, and 100% levels to develop meat products with modified nutritional profiles. The nutritional, physicochemical, and sensory properties of the patties were investigated. The results indicated that as the replacement proportion of AB increased, the patties exhibited higher moisture (from 62.81% to 77.85%), dietary fiber (from 0% to 1.76%), and ash (from 3.27% to 3.73%) content. Concurrently, the fat content decreased from 4.49% to 2.17%, protein fell from 23.79% to 6.70%, and the energy value reduced from 135.57 to 49.67 kcal/100 g). The texture of patties was softened with higher replacement proportion of AB. Sensory evaluation revealed that patties with 25% and 50% replacement proportion of AB received overall acceptability scores of 8.10 and 7.65, respectively, which were not significantly different (*p* > 0.05) from the control (8.25). The results of this work suggest that AB has potential as a substitute for lean pork to modify nutritional profiles, with up to 50% substitution yielding a product with desirable sensory properties reduced fat and increased dietary fiber.

## 1. Introduction

Pork patties are widely popular in the fast-food industry, restaurants, and households due to their high nutritional value, convenience, cost-effectiveness, and appealing flavor. These attributes make pork patties excellent candidates for the development of functional meat products [1,2,3]. However, the excessive consumption of red meat and fat, the main components of pork patties, has been linked to various chronic diseases such as obesity, cardiovascular disease, type 2 diabetes, and gastrointestinal cancer [4,5]. Consequently, there is a pressing need to reduce the content of red meat and fat in pork patties. One promising approach is the use of functional substitutes to partially replace meat. The substitutes can originate from plants, animals, or microbial products [6]. Previous studies have explored the use of plant-based ingredients (e.g., pulses, cereals, tubers and fruits), mushroom, insects and by-products from the food industry as meat alternatives [7,8,9,10]. Common meat substitutes include food fibers, such as tomato pomace, rice bran, non-meat proteins, and hydrocolloids [10]. While the previous studies have successfully achieved partial meat replacement, issues related to texture and sensory quality remain. Therefore, there is a need for a novel, cost-effective meat substitute that can enhance both the sensory characteristics and nutritional value of pork patties.

Edible mushrooms, particularly *Agaricus bisporus* (AB), have emerged as innovative ingredients in plant-based meat product development. AB mushrooms are rich in dietary fiber, essential amino acids, vitamins, and various bioactive compounds such as polysaccharides (e.g., β-glucans) and antioxidants, making it a promising meat substitute alongside plant-based proteins [11,12]. As a widely cultivated and consumed edible basidiomycete, AB constitutes a significant portion of the global edible fungi production, and is associated with improved immune function, cholesterol reduction, and antioxidant activity [13,14,15]. These attributes make promising candidate for developing healthier meat products by partially replacing meat components, potentially contributing to the reduced intake of saturated fats and calories, factors linked to chronic diseases like obesity and cardiovascular disorders [4,5]. Previous studies have explored the use of various mushrooms, including Lentinula edodes (shiitake) and Pleurotus ostreatus (oyster mushroom), as meat extenders or replacers in products like sausages and patties [10,16]. Wang et al. reported successful partial replacement of lean pork with *Lentinula edodes* in sausages, observing improved dietary fiber content and certain sensory attributes, albeit with changes in texture. Vargas-Sanchez et al. demonstrated the antioxidant and antibacterial effects of *Agaricus brasiliensis* extract in pork patties. However, the processing of AB generates a considerable amount of mushroom stalks and residuals as waste, which are often undervalued. Utilizing these by-products not only reduces waste but also contributes to the sustainable development of the food industry. While mushrooms have been explored as meat extenders, studies specifically utilizing the stalks of AB as a direct partial replacement for lean pork in patties are scarce. Therefore, this study specifically employed the stalks of AB, rather than the whole mushroom or mushroom powder, to explore their potential as a novel meat substitute.

Herein, the pork patties with different replacement proportions of AB stalks (25%, 50%, 75%, and 100%) were prepared. The nutritional profile, cooking loss, water holding capacity (WHC), texture, volatile compound content, electronic nose analysis, sensory characteristics and microbial analysis of the patties were assessed. The aim of this work was to explore the feasibility of using AB stalks as a meat substitute in pork patties and contribute to the valorization of mushroom by-products and the development of nutritionally enriched meat alternatives.

## 2. Materials and Methods

### 2.1. Materials

The lean pork and fresh AB were purchased from a local market (Changchun, China). The additives and chemical reagents were obtained from Sichuan Jinshan Pharmaceutical Co., Ltd. (Meishan, China) and Beijing Beihua Co., Ltd. (Beijing, China), respectively.

### 2.2. Preparation of Pork Patties

To fabricate pork patties with different substitution levels of AB for pork, a control group of pure pork patties devoid of AB substitution was designated as C. The substitution percentages of AB for pork were set at 25%, 50%, 75%, and 100%, denoted as P25, P50, P75, and P100, respectively. The formulations for the pork patties are shown in Table 1. The procedure for producing the pork patties entails the following steps:

Initially, fresh AB stalks were vacuum-packed and beaten for 3 min using a beating apparatus. A 0.7% aqueous solution of citric acid was heated to 95 °C, and the AB stalks were submerged for 5 min, followed by immersion in cold water until the temperature decreased to 20 °C. A 0.01% aqueous solution of monascus red water was incorporated into the mixture, which was then sealed and allowed to stand for 15 min. This step was implemented to standardize the color across all formulations to a meat-like hue, minimizing visual bias in subsequent sensory evaluation and focusing panelist assessment on other attributes such as texture and flavor. Subsequently, lean pork and AB were sliced into thin pieces of approximately 2–3 cm. According to the formulations listed in Table 1, the lean pork, AB, and additives were blended and homogenized for 240 s. The resultant mixture was molded into circular shapes with a diameter of 2.5 cm, cooked in a steam-convection oven for 15 min, and then grilled in a parallel-bar electric grill for 2 min (turning the patties once every 30 s). The dual cooking process (steaming followed by grilling) was used to simulate common household and commercial practices, ensuring the patties were fully cooked while achieving a desirable surface color and flavor through Maillard reaction. Finally, the pork patties were cooled to ambient temperature, vacuum-packaged in polyethylene bags using a vacuum packaging machine (Zhucheng Yizhong Machinery Co., Ltd., Zhucheng, China), and stored at 4 ± 1 °C for subsequent analysis. The entire experiment, including patty preparation and all analyses, was independently replicated three times (*n* = 3) on different days using freshly prepared materials.

### 2.3. Analysis of Proximate Composition and Energy Value

The proximate composition analysis was conducted according to the Association of Official Analytical Chemists (AOAC, 2005). The moisture content was determined by oven drying at 105 °C (AOAC 950.46). The crude protein content (N × 6.25) was measured by the Kjeldahl method (AOAC 928.08). The fat content was determined by Soxhlet extraction with petroleum ether (AOAC 960.39). The ash content was determined by incineration in a muffle furnace at 550 °C (AOAC 920.153). The total dietary fiber content was analyzed by the enzymatic-gravimetric method (AOAC 991.43). All determinations were performed in triplicate. The energy value was calculated based on the following standards: 9 kcal/g for fat, 4 kcal/g for protein, 4 kcal/g for carbohydrates, and 2 kcal/g for total dietary fiber (European Union (EU), 2011).

### 2.4. Determination of Water Activity and pH

The water activity of the pork patties was determined using a HygroLab 2 four-channel desktop water activity meter (Rotronic, Bassersdorf, Switzerland). The pH of the pork patties was measured using a portable digital potentiometer (pH meter, Mettler Toledo, Greifensee, Switzerland) with a penetration electrode. The instrument was calibrated using standard pH 4.0 and 7.0 buffers prior to measurements. Each patty was measured in triplicates.

### 2.5. Determination of Cooking Loss and WHC

The cooking loss was investigated using the method described by Wang et al. with slight modifications [10]. Individual raw patties were weighed (50 g) and then cooked at 80 °C for 50 min. The cooking loss was calculated using the following equation:

(1) $$\mathrm{Cooking}\;\mathrm{loss}\;(\%)=\frac{m_{1}-m_{2}}{m_{1}}\times 100\%$$

where *m*_1_ is the weight of raw pork patties and *m*_2_ is the weight of cooked pork patties.

Each sample was centrifuged at 12,000× g for 30 min at 4 °C. The WHC was calculated as a percentage of retained water using the following formula:

(2) $$\mathrm{WHC}\;(\%)=\frac{w_{2}}{w_{1}}\times 100\%$$

where *w*_1_ is the weight of sample before centrifugation, and *w*_2_ is the weight of the sample after centrifugation.

### 2.6. Color Measurement

The external and internal color of the patties was analyzed using a HunterLab ColorFlex (illuminant: D65, standard observer: 10, Xinlian Creation Electronic Co., Ltd., Shanghai, China). The aperture of the meter was 14 mm. The color parameters of L* (lightness), a* (negative—green; positive—red), and b* (negative—blue; positive—yellow) were measured. A standard plate CX 2064 (L* = 52.08, a* = −26.39, b* = 14.88) was used as a reference. The total color difference (∆E*) between each sample and the control (C) was calculated using the following formula [10]:

(3) $$∆E=(∆a^{2}+∆b^{2}+∆L^{2}{)}^{\frac{1}{2}}$$

where ∆*a* = a* − a*_sample_, ∆*b* = b* − b*_sample_, ∆*L* = L* − L*_sample_.

### 2.7. Texture Profile Analysis (TPA)

The TPA was conducted on pork patties using a texture analyzer (Model: TMA-Pro, Manufacturer: FTC, Sterling, VA, USA), with 5 replicates per sample. Cylindrical cuts of 2 cm in diameter and 1 cm in height were prepared from the central portion of each sample group. The samples underwent a two-bite compression test using a cylindrical probe. The test settings were as follows: a pre-test, test, and post-test speed of 6 cm/min, a strain of 50% of the original sample height, and a pause of 20 s between the two compressions. Results were reported as the average of at least 10 repeatable runs per test procedure. The hardness (N), springiness (m), cohesiveness (Ns), chewiness, and gumminess of the pork patties were evaluated.

### 2.8. Determination of Amino Acid Content

The amino acid content was determined using an external standard calibration curve with known concentrations of amino acid standards described by Reis et al. with minor modifications [16]. Minced pork patties (100 mg) were weighed into a sealed bottle, and 10 mL of 6 mol/L hydrochloric acid (containing 1% phenol) was added. The bottle was filled with nitrogen for 1 min, sealed, and hydrolyzed at 110 °C for 22 h. After cooling, the reactant was diluted to 50 mL with water. Then, 1 mL of diluted reactant was taken and dried under nitrogen at 95 °C, accurately dissolved in 1 mL 0.01 mol/L HCl, and filtered for measurement. The measurement conditions were as follows: Agilent 1100 liquid chromatograph (equipped with VWD detector); chromatographic column: ZORBAX Eclipse AAA (4.6 × 75 mm, 3.5 μm); detection signal: UV at 338 nm (0–19 min) and 266 nm (19.01–25 min); mobile phase A: 40 mM sodium dihydrogen phosphate (pH 7.8); mobile phase B: acetonitrile/methanol/water = 45/45/10; flow rate: 1.0 mL/min. The chemical score (CS) was calculated as follows: CS = (mg of amino acid in 1 g of test protein/mg of amino acid in 1 g of reference protein) × 100, using the FAO/WHO/UNU amino acid reference pattern for adults [17].

### 2.9. Electronic-Nose (E-Nose)

E-nose analysis was conducted using the method described by Yin et al. with minor modifications [15]. The PEN3 electronic nose (Airsense, Schwerin, Germany) was calibrated according to the instructions of manufacturer using standard gases, which contains 10 metal oxide sensors. Sample processing involved cutting different pork patties into suitable small patties, placing 5 samples in 5 headspace bottles, covering them with plastic wrap, and allowing them to stand at room temperature for 1 h (to allow full volatilization of flavors). The parameters were set as follows: sampling time interval of 1 s per group, automatic sensor cleaning time of 100 s, sensor zeroing time of 5 s, sample flow rate of 200 mL/min, and experimental test analysis time of 100 s. Each sample was repeated three times.

### 2.10. Volatile Compound

The volatile compound content was determined using the method designed by Yin et al. [18]. Briefly, minced pork patties (4 g) were placed in a 20 mL headspace vial, and 4 μL of 1,2-dichlorobenzene (100 mg/L in methanol) was added as an internal standard to correct for variations in sample preparation and instrument response. The vial was sealed with a Teflon septum. Headspace solid-phase microextraction (HS-SPME) was used to extract volatile compounds, using a 50/30 μm DVB/CAR/PDMS (divinylbenzene/carboxen/polydimethylsiloxane) fiber. The compound was extracted at 50 °C and equilibrated for 30 min at the same temperature. with agitation, followed by extraction at the same temperature for an additional 30 min. The adsorbed compounds were then desorbed in the GC injector at 250 °C for 3 min in non-fragmentation mode. Gas chromatography-mass spectrometry (GC-MS) was used for identification (Agilent 5975-6890N, Palo Alto, CA, USA). A capillary column with inert cap wax (0.25 mm × 0.25 μm × 60 m) was used to separate volatile compounds. Helium was used as the carrier gas, with a column flow rate of 1.5 mL/min. The oven temperature was maintained at 40 °C for 2 min, then increased to 90 °C at 3 °C/min, followed by an increase to 200 °C at 3 °C/min, held at 200–230 °C for 15 min, and finally held for an additional 10 min. The MS operated in scan mode within the range of 45–450 *m*/*z*, with electron ionization performed at 70 eV.

Volatile compounds were identified by comparing their mass spectra with the NIST library and by calculating their retention indices relative to a standard mixture of n-alkanes (C7–C30). For semi-quantitative analysis, the peak area of each volatile compound was integrated and normalized to the peak area of the internal standard (1,2-dichlorobenzene). The relative content of each compound is expressed as a percentage of the total normalized peak area of all identified volatiles (relative percentage area, %).

### 2.11. Sensory Characteristics

The sensory evaluation of patties was conducted using the method described by Jin et al. with minor modifications [19]. The evaluation panel consisted of 15 researchers, 5 technicians, and 10 graduate students from Jilin Agricultural University (50% male/female, aged 20 to 60). The sample size of 30 panelists was selected based on previous study in meat products [10]. All panelists were trained and familiar with the attributes being evaluated. The evaluation was conducted in 3 rounds, with a total of 15 evaluations per round. Each time, three pork patty samples were provided to panel members for five evaluations each. The pork patties were cooked, cooled to room temperature, and then cut into cubes of 1 cm in length, width, and height. The samples were served warm (approximately 60 °C) on white plastic plates, and labeled with 3 random digits. Water and unsalted biscuits were also provided to the panel members for taste cleansing. The sensory test used a 10-point scale (10 = extremely desirable; 1 = extremely undesirable), based on the intensity of key attributes: appearance, aroma, texture, flavor, and overall acceptability. Product acceptance was calculated as the total score of five indicators divided by 5.

### 2.12. Determination of Microbiological Quality

Each pork patty (25 g) was homogenized in 225 mL of sterile 0.1% peptone buffer for 2 min using a Stomacher laboratory blender. Appropriate decimal dilutions were then spread onto selective agar plates. Total viable counts (TVC) were determined on Plate Count Agar at 37 °C for 48 h. Coliform and Staphylococcus aureus were detected using Violet Red Bile Agar and Baird–Parker Agar, respectively, incubated at 37 °C for 48 h. For mold cultivation, the samples were spread onto Potato Dextrose Agar and incubated at 25 °C for 72 h, according to the method described by Siripatrawan et al. [20].

### 2.13. Statistical Analysis

All analyses were performed with three independent batches (*n* = 3) of patties prepared on different days. Data were expressed as mean value ± standard deviation (SD) of these three independent replicates. Statistical analysis was carried out using one-way analysis of variance (ANOVA) and Duncan’s multiple comparing test through SPSS software (Version 23.0, SPSS Inc., Chicago, IL, USA). Statistical significance was set at *p* < 0.05. For the analysis of sensory data, the pork patties type was used as a fixed factor (5 levels), and panelists (30 judges) were used as a random factor.

## 3. Results and Discussion

### 3.1. Proximate Composition and Energy Value Analysis

The results of proximate composition and energy value analysis of the pork patties are shown in Table 2. With the increase in the replacement proportion of AB, the fat content of the replacement groups (P25, P50, P75 and P100) significantly decreased by 0.13%, 0.36%, 0.43%, and 0.52%, respectively, compared with the control group, which was consistent with the results reported by Baune et al. [21]. In Baune’s study, adding other ingredients to replace meat resulted in a reduction in the fat content of the meatballs. The control group had the highest protein content at 23.79%, but as the AB replacement proportion increased from 25% to 100%, the protein content decreased significantly, from 21.88% to 6.7%, which was consistent with the results reported by Gao et al. [22]. The significant decrease in protein content with AB substitution may affect the nutritional quality of the patties. However, the increase in dietary fiber and essential amino acids such as methionine partially compensates for this reduction. From a functional perspective, the decline in protein content contributed to the softer texture observed in TPA, as myofibrillar protein network integrity was compromised [23].

The replacement proportion of AB had a significant effect on the water content of pork patties (*p* < 0.05). The water content of control group was 62.81%. Compared with the control group, the water content of P25, P50, P75 and P100 increased by 1.9%, 7.74%, 11.57% and 15.04%, respectively. This can be attributed to the addition of AB, which has a higher water content [24]. In addition, AB as a type of dietary fiber can retain water [25].

The replacement proportion of AB had a significant effect on the dietary fiber of pork patties (*p* < 0.05). With the increase in AB, dietary fiber increased from 0% to 1.67%, which was similar to that reported by Choi et al. [26].

The ash content of the control group was 3.27%. When the replacement proportion of AB was 25%, the ash content of pork patties was 3.33%, which was not significantly different from that of control group (*p* > 0.05). With the replacement proportion of AB increasing to 50%, 75% and 100%, the ash content of pork patties began to increase significantly to 3.46%, 3.52% and 3.73% (*p* < 0.05).

The energy value of the control group was 135.57. As the replacement proportion of AB increased, the energy of the pork patties decreased significantly (*p* < 0.05), from 122.61 at 25% AB replacement proportion to 49.67% at 100% AB replacement proportion. This is because the fat and protein of the pork patties were declined, which in turn led to a decrease in the energy value of the pork patties.

### 3.2. Water Activity and pH

The water activity and pH value of pork patties are evaluated in Table 2. The replacement of AB for lean pork in pork patties did not result in a significant change in water activity. The pH of pork patties in the control group was 6.31. As the proportion of AB replacing lean pork increased, the pH value of pork patties showed an upward trend. This may be because the pH of the AB was higher than that of lean pork, causing the pH of the pork patties to rise with the increase in the replacement proportion of AB.

### 3.3. Cooking Loss and WHC

The cooking loss and water holding capacity of pork patties are shown in Table 3. The cooking loss can be demonstrated by the level of exudate and fat loss. With the increase in the replacement proportion of AB, the cooking loss rate of pork patties increased significantly (*p* < 0.05). This may be due to the increased replacement proportion of AB leading to water and fat loss, causing the muscle protein denaturation [27]. With the increase in replacement proportion of AB, the WHC of pork patties in the replacement groups (P25, P50, P75 and P100) decreased by 2.03%, 6.25%, 11.95% and 19.84%, respectively. This is possibly because the addition of AB could disrupt the structure of the pork patties, making the WHC of the pork patties decrease. The reduction in WHC and increase in cooking loss may also be attributed to the disruption of the protein matrix by AB fibers, which interfere with water binding and fat retention. Similar findings have been reported in studies incorporating dietary fibers from other sources [28]. Furthermore, a similar trend was observed by Wang et al., who reported increased cooking loss in sausages with higher levels of Lentinula edodes substitution, which was also attributed to the disruption of the protein matrix and the inherent differences in water-binding capacity between meat and mushroom components [10].

Compared to studies incorporating mushroom powder, the use of intact AB stalks in this study may offer distinct advantages [29]. The fibrous and cellular structure of the stipes could contribute to a meat-like texture and water-holding capacity in a different manner than finely ground powder, which primarily acts as a filler. Furthermore, utilizing the minimally processed stipe directly aligns with a clean-label trend and reduces processing energy costs, enhancing the sustainability of the proposed approach.

### 3.4. TPA

The texture properties of pork patties are highly correlated with the properties of myofibrils [30]. The TPA parameters of pork patties are listed in Table 3. With the increase in the replacement proportion of AB, TPA parameters significantly decreased (*p* < 0.05), indicating that AB reduced the hardness, cohesion, viscosity, chewiness, and elasticity of the pork patties. This was primarily due to the soft texture of hot scalded AB. The results of TPA were consistent with the previous study [31]. The hardness of pork patties was associated with protein content. With the increase in the replacement proportion of AB, the protein content decreased, leading to a decrease in hardness. There was no significant difference in cohesion between the replacement group of P25 and the control group (*p* > 0.05), indicating that P25 in the replacement group was most similar to the control group.

### 3.5. Color Characteristics

**Figure 1:**
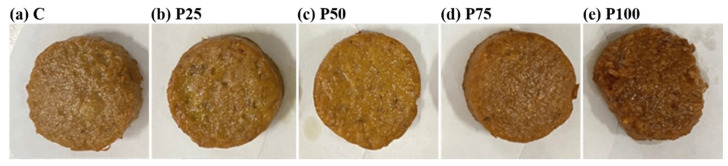
Images of pork patties with AB in different replacement proportion.

Figure 1 shows pork patties with AB in different replacement proportions. As the replacement proportion of AB increased, the pork patties gradually deepened in color. Table 4 shows the effects of AB replacement proportion on the L*, ∆E*, a* and b* values of pork patties. In terms of external color, with the increase in the replacement proportion of AB, the L* values of pork patties decreased, and the ∆E* values increased significantly. There was no significant difference between a* and b* value, but the L* value of meat emulsion decreased with the increasing replacement proportion of AB, which was consistent with the previous study [32]. The change in Table 4 indicates that the color of pork patties became slightly darker as the replacement proportion of AB increased, which could be attributed to the fact that AB was not as bright as pork. In addition, there was no significant difference between a* and b* values, suggesting that AB had strong similarity to pork after staining with monascus red.

In terms of internal color, L* and ∆E* exhibited the same change trend as L* and ∆E* of external color. The a* value increased from 8.30 to 8.91, but there was no significant difference between P25 and P50 in the replacement group. The b* value showed an overall upward trend, but P25 had no significant difference compared with the control group. The external colors had a higher a* values and lower b*, ∆E* and L* values compared to the internal colors due to frying during processing.

### 3.6. Amino Acid Profile

Amino acids are important substances for maintaining human life activities, and AB is a significant source of amino acids [33]. As shown in Table 5, amino acid content of the patties was influenced by the replacement proportion of AB. Compared to the control group, the methionine concentration increased significantly (*p* < 0.05) with increasing replacement proportion of AB stalks (P25, P50, P75 and P100). In contrast, a general declining trend was observed for other essential amino acids (EAAs) such as valine, lysine, leucine, isoleucine, and threonine. Among non-essential amino acids (NEAAs), serine and glutamate remained relatively stable, while the concentrations of histidine, glycine, aspartic acid, tyrosine, alanine, proline, and cysteine decreased as the replacement level increased.

The total EAA content exhibited a significant decreasing trend from 87.38 g/100 g sample in the control to 32.16 g/100 g sample in P100, concomitant with the reduction in crude protein, a finding consistent with previous study [10]. To comprehensively evaluate the impact on protein nutritional quality, the chemical score (CS) was calculated against the FAO/WHO/UNU amino acid reference pattern for adults [32]. This analysis revealed a critical shift in protein quality. The control (C) and P25 groups exhibited a well-balanced amino acid profile, with all EAAs exceeding the reference requirements (CS > 100%). However, for patties with ≥50% substitution, a significant nutritional limitation emerged. Lysine became the first limiting amino acid in groups P50, P75, and P100, with its CS plummeting to approximately 79, 54, and 15, respectively. Threonine and leucine also showed increasing deficiency at high substitution levels. This indicates that while AB contributes beneficial methionine, its overall EAA profile is inferior to that of lean pork, particularly regarding lysine content. Consequently, the increase in methionine could not compensate for the severe deficit of lysine. Therefore, while partial substitution (25%) maintains a high-quality protein profile, high-level substitution (≥50%) substantially compromises the protein quality, resulting in a product that would be inadequate as a sole source of dietary protein without fortification or complementary dietary sources.

### 3.7. E-Nose Analysis

**Figure 2:**
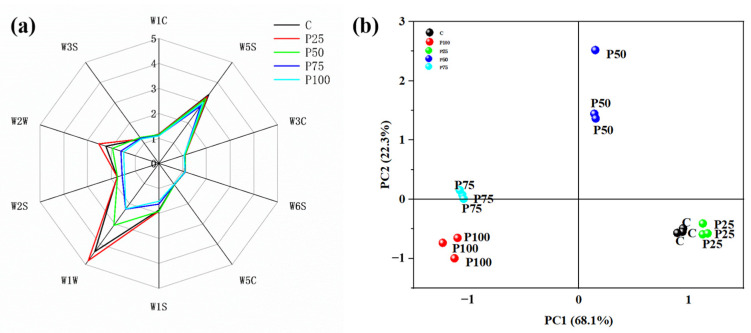
Radar plot (**a**) and PCA (**b**) of pork patties with AB in different replacement proportions.

The radar plot shown in Figure 2a demonstrates the response intensity of various sensors to the volatile compounds in different pork patties. Compared to sensors W1C, W3C, W6S, W5C, and W3S, sensors W5S, W1W, W2W, W1S, and W2S exhibited significantly enhanced and distinctive responses to the pork patties, indicating that the pork patties may contain higher concentrations of sulfur compounds, alcohols, aldehydes, ketones, and aromatic compounds. The meaning of indicators such as W1C is shown in Table 6. Compared with the control group, the replacement group of P25 exhibited relatively high response values on sensors W1W, W2W, and W1S, indicating that P25 pork patties may contain more abundant sulfur compounds and aromatic compounds, which play crucial roles in flavor formation. The heightened response of sensors W1W and W1S, indicating sulfur and aromatic compounds, aligns with the known flavor profile of mushrooms as reported by Selli et al. [34].

The principal component analysis (PCA) is a statistical tool used to explain differences between samples by extracting information from variables that primarily affect the spatial distribution of samples. There are usually two extraction criteria: the extraction is greater than 1 and the total contribution variance is greater than 80% [35]. Figure 2b shows the PCA results of pork patties with different replacement proportion of AB. The first two principal components collectively account for 90.4% of the total variance, thereby encompassing the majority of the information related to the odors of the pork patties. Specifically, the first principal component (PC1) explained 68.1% of the variance, showing a positive correlation with the replacement group of P75 and P100, and a negative correlation with the replacement group of P25 and P50. The second principal component (PC2) accounts for 22.3% of the variance, exhibiting a negative correlation with the replacement group of P25 and P100, and a positive correlation with the replacement group of P75 and P50. The control group and the replacement groups P25 and P50 were positioned on the right side of the X-axis, whereas the other replacement groups P75 and P100 were located on the left side. This grouping aligned with the outcomes of the radar plot, further confirming the prominence of sulfur compounds, alcohols, aldehydes, ketones, and aromatic compounds in the odors of the pork patties. Notably, the replacement group of P25 contained more sulfur compounds and aromatic compounds, a conclusion that was consistent with Figure 2a.

### 3.8. Volatile Compound Analysis

Due to the complexity and cost of the analysis, the volatile compound profiling was conducted on a representative sample per treatment to identify key trends and compositional changes. Therefore, the data in Table 7, Table 8, Table 9, Table 10, Table 11, Table 12, Table 13 and Table 14 should be interpreted for their qualitative and semi-quantitative value. The observed trends are supported by the electronic nose analysis. The volatile compounds in pork patties with different replacement proportion of AB were analyzed using gas chromatography-mass spectrometry (GC/MS) to gain insights into the impact of AB flavor on pork patties. The results of Table 7 revealed a total of 62 volatile compounds detected and semi-quantified across the various pork patties. These compounds encompassed a wide range of chemical classes, including aldehydes, alcohols, ketones, acids, esters, alkenes, alkanes, and others. In control group of pork patties, 35 volatile compounds were identified, while 37, 34, 33 and 21 volatile compounds were identified in the replacement group of P25, P50, P75, and P100, respectively. Aldehydes produced mainly through lipid oxidation and often play an important role in meat flavor, were significantly present [36]. With the increase in AB replacement proportion, the total concentration of aldehyde increased. Specifically, compounds such as Nonanal, Decanal, Benzaldehyde, and 2,4-Decadienal, (E, E)- exhibited notable concentrations, and their levels generally increased with higher replacement proportion of AB. There were 8 alcohols were detected, such as 1-Octen-3-ol, 1-Hexanol, 2-ethyl-, and Benzyl alcohol. No benzyl alcohol was detected in the control group pork patties, but the concentration of benzyl alcohol in replacement groups increased significantly with the increase in replacement proportion of AB. The rise in benzyl alcohol concentration could be attributed to its status as the main volatile compound in AB [34]. Furfuryl alcohol, one of the most abundant furan derivatives, was found in replacement group of P75. In addition, 7 ketones were detected, which were primarily generated through lipid oxidation, amino acid decomposition, and exposure to wood smoke [37]. There were 9 acids were detected, possibly formed by hydrolysis of triglycerides and oxidation of aldehydes. With the increase in AB replacement proportion, the acid concentration in the pork patties decreased, which may be due to the fat content in the pork patty decreased with the increase in AB addition. Moreover, 10 olefins were also detected in the pork patties, which may be related to the addition of spices [38]. At the same time, 4 esters and 10 alkanes were detected. Compounds such as Nonanal, Decanal, and Benzaldehyde contribute to fatty, green, and almond-like notes, respectively, which are desirable in meat products. The increase in benzyl alcohol, a characteristic volatile of AB, may impart a mild fungal aroma, which was only perceptible in the P100 group as noted in sensory evaluation.

### 3.9. Sensory Analysis

**Figure 3:**
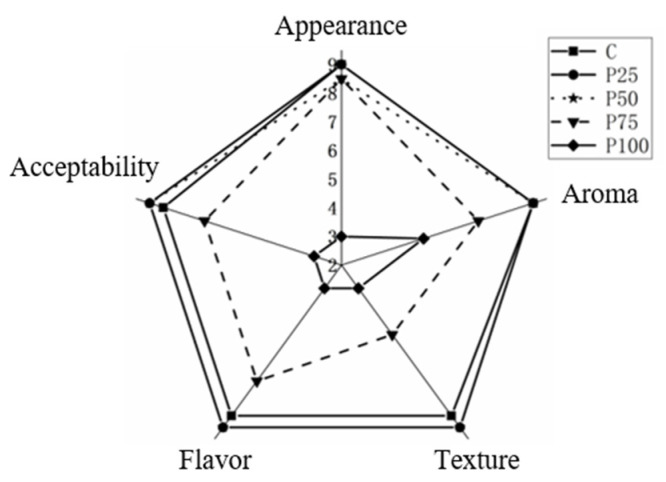
Sensory evaluation of pork patties with varying replacement proportions of AB.

The result of the sensory evaluation of pork patties were presented in Figure 3. In terms of texture, the sensory scores of the control group and the replacement groups (P25 and P50) were higher than the other replacement groups (P50 and P100). This was attributed to the appropriate replacement of pork with AB, which imparted an optimal level of elasticity and firmness to the pork patties. However, excessive replacement of pork with AB resulted in pork patties that were too loose and inelastic, characteristics that were not favored by consumers. Regarding taste, the replacement groups P25, P50 and P75 scored above 7, with P25 achieving the best score. The inherent odor of AB was minimal, and the use of hot soup processing eliminated their native aroma, making it difficult to detect their flavor. Only the replacement group P100 had a slight taste of AB, leading to a lower score. However, pork patties with a high content of AB could be favored by vegetarians. In terms of appearance, there were no significant differences (*p* > 0.05) in the appearance of control group and the replacement group of P25, P50 and P75. The replacement group of P100 exhibited a loose and unformed structure due to the softening of AB after blanching and the inability of their proteins to form myofibrillar protein fibers. In terms of aroma, the sensory score of the replacement group of P100 significantly decreased (*p* < 0.05) compared to the other groups, as the pork patties had a slight taste of AB due to their high content. There were no significant differences (*p* > 0.05) between the control group and the replacement groups of P25, P50, and P75. Statistical analysis revealed no significant differences (*p* > 0.05) in overall acceptability between the control, P25, and P50 groups, although texture and flavor scores decreased slightly with increasing AB substitution. This suggests that up to 50% AB substitution is sensorially acceptable despite slight changes in texture and flavor. P100 was rated as unacceptable and needed improvement. The results of sensory analysis indicate that AB can be used as a substitute for lean pork in pork patties. The results align with previous research indicating that sensory attributes are a primary determinant of product acceptance [39]. The comparable overall acceptability scores of patties with 25% and 50% AB substitution to the control suggest that these products possess the necessary sensory quality to be potentially well-received by consumers, providing a promising approach for reducing fat content without severely compromising sensory appeal.

### 3.10. Microbiological Analysis

The microorganism content of the pork patties is shown in Table 15. The population for total viable counts of control group was 1.35 log CFU/g. The populations for total viable counts of replacement groups were 1.65, 1.15, 0.85 and 1.55 log CFU/g, respectively. No molds, coliforms, Staphylococcus, or Staphylococcus aureus were detected in any of the pork patties. The results indicate that substituting AB for pork have no significant impact on the microbial quality of the pork patties (*p* > 0.05).

## 4. Conclusions

Pork patties with *Agaricus bisporus* (AB) stalks substituting lean pork at levels of 0%, 25%, 50%, 75%, and 100% have been prepared. The incorporation of AB significantly modified the nutritional and physicochemical properties of the patties. The fat content was reduced by up to 51.7% and the energy value was reduce by 63.4% in the 100% substitution group compared to the all-pork control. Furthermore, the dietary fiber content increased from not detected to 1.76%, and the moisture and ash content also rose significantly with higher AB levels. The amino acid profile was altered, with a marked increase in methionine content. The volatile compound analysis revealed an enrichment in aldehydes, alcohols, and alkenes, contributing to a more complex flavor profile, as supported by electronic nose analysis. However, the protein content decreased substantially, and the textural properties (hardness, chewiness, springiness) were significantly softened as AB proportion increased. The color of the patties also darkened with higher substitution levels. Sensory evaluation indicated that patties with 25% and 50% AB substitution maintained comparable overall liking to the all-pork control, with no significant differences in overall acceptability. In contrast, patties with 75% and 100% substitution were less acceptable. This study demonstrated that AB stalks can effectively function as a partial substitute for lean pork, primarily creating a meat product with a modified nutritional profile, characterized by reduced fat and increased dietary fiber content. However, it also led to a substantial decrease in protein content and altered the amino acid profile, with lysine becoming the limiting amino acid at substitution levels above 50%. Therefore, a substitution level of up to 50% is recommended to achieve a favorable balance between nutritional modulation, acceptable physicochemical properties, sensory preference, and the maintenance of adequate protein quality. While this study focused on compositional changes, future research should include analysis of lipid oxidation products and fatty acid profiles to provide a more comprehensive assessment of the health-related aspects of AB substitution in meat products. Exploring the application of AB stalks in other meat products also holds promise.

## Figures and Tables

**Table 1:** Formulations of patties with pork lean meat replaced by AB.

Ingredients (%)	C	P25	P50	P75	P100
Pork lean meat	76	57	38	19	0
Stalk of *Agaricus bisporus*	0	19	38	57	76
Additives					
Salt	1.15	1.15	1.15	1.15	1.15
Sugar	0.75	0.75	0.75	0.75	0.75
Monosodium glutamate	0.75	0.75	0.75	0.75	0.75
Sodium pyrophosphate	0.1	0.1	0.1	0.1	0.1
Sodium tripolyphosphate	0.05	0.05	0.05	0.05	0.05
Vitamin C	0.4	0.4	0.4	0.4	0.4
White pepper	0.2	0.2	0.2	0.2	0.2
Carrageenan	0.05	0.05	0.05	0.05	0.05
Isolated soy protein	3	3	3	3	3
Dry starch	5	5	5	5	5
Sodium pyrophosphate	0.1	0.1	0.1	0.1	0.1
Soy sauce	0.75	0.75	0.75	0.75	0.75
Ice	11.8	11.8	11.8	11.8	11.8
Total	100	100	100	100	100

C, P25, P50, P75, and P100 represent 0%, 25%, 50%, 75%, and 100% substitution of pork lean meat by AB, respectively.

**Table 2:** Proximate Composition of pork patties (mean ± standard error).

Parameters	C	P25	P50	P75	P100
Fat (%)	4.49 ± 0.26 ^a^	3.89 ± 0.29 ^b^	2.86 ± 0.13 ^c^	2.54 ± 0.71 ^c^	2.17 ± 0.25 ^d^
Protein (%)	23.79 ± 0.30 ^a^	21.88 ± 0.13 ^b^	16.81 ± 0.26 ^c^	11.63 ± 0.13 ^d^	6.70 ± 0.22 ^e^
Moisture (%)	62.81 ± 0.64 ^e^	64.71 ± 0.45 ^d^	70.55 ± 0.46 ^c^	74.38 ± 0.30 ^b^	77.85 ± 0.07 ^a^
TDF (%)	ND	0.05 ± 0.01 ^a^	0.08 ± 0.01 ^b^	1.32 ± 0.04 ^c^	1.76 ± 0.05 ^d^
Energy value (%)	135.57 ^a^	122.61 ^b^	93.14 ^c^	71.94 ^d^	49.67 ^e^
Ash (%)	3.27 ± 0.02 ^d^	3.33 ± 0.03 ^d^	3.46 ± 0.03 ^c^	3.52 ± 0.02 ^b^	3.73 ± 0.03 ^a^
Water activity	0.97 ± 0.00 ^a^	0.97 ± 0.00 ^a^	0.97 ± 0.01 ^a^	0.97 ± 0.01 ^a^	0.97 ± 0.01 ^a^
pH	6.31 ± 0.02 ^e^	6.36 ± 0.01 ^d^	6.38 ± 0.05 ^c^	6.53 ± 0.15 ^b^	6.79 ± 0.00 ^a^

^a–e^: Statistical differences between different sample groups are shown with different letters (*p* < 0.05). ND: not determined. C, P25, P50, P75, and P100 represent 0%, 25%, 50%, 75%, and 100% substitution of pork lean meat by AB, respectively.

**Table 3:** Texture profiles of pork patties (mean ± standard error).

Parameters	C	P25	P50	P75	P100
Cooking loss (%)	11.42 ± 0.15 ^d^	12.50 ± 0.16 ^c^	15.00 ± 0.11 ^b^	15.82 ± 0.93 ^b^	16.95 ± 0.15 ^a^
Water holding capacity (%)	89.51 ± 0.22 ^a^	87.48 ± 0.20 ^b^	83.26 ± 0.06 ^c^	77.56 ± 0.07 ^d^	69.67 ± 0.17 ^e^
Hardness (N)	104.97 ± 2.81 ^a^	76.80 ± 1.39 ^b^	53.0 ± 6.68 ^c^	18.40 ± 0.46 ^d^	3.30 ± 0.30 ^e^
Cohesiveness	0.50 ± 0.02 ^a^	0.47 ± 0.01 ^a^	0.34 ± 0.02 ^b^	0.32 ± 0.02 ^b^	0.22 ± 0.02 ^c^
Gumminess (N)	78.53 ± 0.70 ^a^	67.33 ± 0.80 ^b^	56.43 ± 0.25 ^c^	52.90 ± 0.20 ^d^	2.73 ± 0.05 ^e^
Chewiness (mJ)	201.85 ± 10.94 ^a^	100.95 ± 0.55 ^b^	32.43 ± 2.65 ^c^	11.58 ± 1.34 ^d^	0.57 ± 0.02 ^e^
Springiness (mm)	6.51 ± 0.11 ^a^	6.43 ± 0.01 ^a^	5.39 ± 0.22 ^b^	3.70 ± 0.02 ^b^	0.22 ± 0.02 ^c^
Cooking loss (%)	11.42 ± 0.15 ^d^	12.50 ± 0.16 ^c^	15.00 ± 0.11 ^b^	15.82 ± 0.93 ^b^	16.95 ± 0.15 ^a^

^a–e^: Statistical differences between different sample groups are shown with different letters (*p* < 0.05). ND: not determined. C, P25, P50, P75, and P100 represent 0%, 25%, 50%, 75%, and 100% substitution of pork lean meat by AB, respectively.

**Table 4:** The external & inter color L*, a* and b* values of pork patties (mean ± standard error).

	C	P25	P50	P75	P100
External color					
L*	44.44 ± 0.08 ^a^	44.31 ± 0.28 ^a^	43.44 ± 0.08 ^b^	42.45 ± 0.46 ^c^	40.57 ± 0.20 ^d^
a*	12.58 ± 0.03 ^ab^	12.48 ± 0.06 ^bc^	12.41 ± 0.11 ^c^	12.69 ± 0.06 ^a^	12.54 ± 0.09 ^abc^
b*	24.40 ± 0.14 ^a^	22.50 ± 0.21 ^d^	22.60 ± 0.08 ^cd^	22.76 ± 0.02 ^bc^	22.92 ± 0.02 ^b^
∆E*	ND	1.91 ± 0.08 ^d^	2.07 ± 0.13 ^c^	2.58 ± 0.05 ^b^	4.14 ± 0.16 ^a^
Inter color					
L*	56.04 ± 0.04 ^a^	53.51 ± 0.03 ^b^	52.60 ± 0.09 ^c^	49.45 ± 0.23 ^d^	48.32 ± 0.25 ^e^
a*	8.30 ± 0.01 ^d^	8.54 ± 0.04 ^c^	8.64 ± 0.06 ^c^	8.80 ± 0.06 ^b^	8.91 ± 0.05 ^a^
b*	23.55 ± 0.08 ^c^	23.50 ± 0.03 ^c^	23.28 ± 0.16 ^d^	24.45 ± 0.06 ^b^	24.95 ± 0.02 ^a^
∆E*	ND	2.54 ± 0.05 ^d^	4.00 ± 0.08 ^c^	6.67 ± 0.14 ^b^	7.87 ± 0.12 ^a^

^a–e^: Statistical differences between different sample groups are shown with different. ND: not determined. C, P25, P50, P75, and P100 represent 0%, 25%, 50%, 75%, and 100% substitution of pork lean meat by AB, respectively.

**Table 5:** Amino Acid Content (g/100 g sample, wet basis).

Parameters	C	P25	P50	P75	P100
Essential amino acid (g/100 g)					
Valine	17.50 ± 0.06 ^a^	10.45 ± 0.04 ^b^	11.07 ± 0.02 ^c^	7.65 ± 0.03 ^d^	2.60 ± 0.08 ^e^
Methionine	5.10 ± 0.04 ^a^	5.14 ± 0.06 ^b^	5.72 ± 0.03 ^c^	6.10 ± 0.07 ^d^	6.26 ± 0.02 ^e^
Lysine	17.83 ± 0.03 ^a^	10.02 ± 0.04 ^b^	10.19 ± 0.03 ^c^	6.95 ± 0.03 ^d^	1.88 ± 0.01 ^e^
Isoleucine	9.12 ± 0.14 ^a^	5.31 ± 0.05 ^b^	5.71 ± 0.08 ^c^	4.01 ± 0.07 ^d^	1.35 ± 0.13 ^e^
Leucine	17.49 ± 0.08 ^a^	10.45 ± 0.07 ^b^	11.07 ± 0.09 ^c^	7.65 ± 0.08 ^d^	2.60 ± 0.10 ^e^
Phenylalanine	9.53 ± 0.07 ^b^	9.73 ± 0.05 ^a^	9.12 ± 0.03 ^c^	8.49 ± 0.05 ^d^	8.93 ± 0.04 ^e^
Threonine	10.21 ± 0.10 ^a^	9.89 ± 0.03 ^b^	9.32 ± 0.08 ^c^	8.35 ± 0.10 ^d^	8.54 ± 0.06 ^e^
Total EAA	86.78 ± 0.52 ^a^	60.99 ± 0.34 ^b^	62.20 ± 0.36 ^c^	49.20 ± 0.43 ^d^	32.16 ± 0.44 ^e^
Non-essential amino acid (g/100 g)					
Histidine	7.71 ± 0.03 ^a^	5.72 ± 0.04 ^b^	4.52 ± 0.02 ^c^	2.88 ± 0.06 ^d^	0.72 ± 0.07 ^e^
Serine	9.26 ± 0.07 ^b^	9.52 ± 0.12 ^a^	8.94 ± 0.02 ^d^	9.06 ± 0.14 ^c^	8.87 ± 0.13 ^e^
Arginine	16.42 ± 0.08 ^a^	16.43 ± 0.18 ^a^	15.89 ± 0.05 ^c^	15.36 ± 0.09 ^b^	14.95 ± 0.08 ^d^
Glycine	8.44 ± 0.02 ^a^	4.99 ± 0.01 ^b^	5.53 ± 0.06 ^c^	3.65 ± 0.03 ^d^	1.31 ± 0.02 ^e^
Aspartic	20.85 ± 0.11 ^a^	11.52 ± 0.08 ^b^	12.10 ± 0.10 ^c^	9.09 ± 0.08 ^d^	3.58 ± 0.09 ^e^
Glutamic	36.63 ± 0.04 ^a^	37.54 ± 0.09 ^b^	36.69 ± 0.11 ^a^	37.18 ± 0.15 ^c^	37.56 ± 0.17 ^b^
Tyrosine	7.76 ± 0.14 ^a^	4.57 ± 0.06 ^b^	4.79 ± 0.05 ^c^	3.39 ± 0.04 ^d^	1.20 ± 0.03 ^e^
Alanine	12.19 ± 0.04 ^a^	7.58 ± 0.03 ^b^	7.69 ± 0.07 ^c^	5.07 ± 0.05 ^d^	1.83 ± 0.04 ^e^
Proline	7.85 ± 0.02 ^a^	3.05 ± 0.07 ^b^	3.33 ± 0.06 ^c^	3.50 ± 0.04 ^d^	1.05 ± 0.07 ^e^
Cysteine	0.35 ± 0.06 ^a^	0.32 ± 0.08 ^a^	0.26 ± 0.01 ^b^	0.15 ± 0.07 ^c^	0.08 ± 0.09 ^d^
Total NEAA	127.46 ± 0.61 ^a^	101.24 ± 0.76 ^b^	99.74 ± 0.55 ^c^	89.33 ± 0.75 ^d^	71.15 ± 0.79 ^e^

^a–e^: Statistical differences between different sample groups are shown with different letters (*p* < 0.05). C, P25, P50, P75, and P100 represent 0%, 25%, 50%, 75%, and 100% substitution of pork lean meat by AB, respectively.

**Table 6:** Meaning of e-nose indicators.

Sensor Name	Responsive Substance	Performance Description
W3C W6S	Aromatic Hydrogen	Sensitive aroma, ammonia Mainly selective for hydrides
W5C W1S W1W W2S W2W W3S	Arom-aliph Broad-methance Sulfur-alcohol Broad-alcohol Sulph-chlor Methane-aliph	Short-chain alkance aromatic component Sensitive to methyl Sensitive to sulfides Sensitive to alcohols, aldehydes and ketones Aromatic ingredients, sensitive to organic Sulfides Sensitive to long-chain alkanes
W1C	Aromatic	Aromatic costituents, benzene
W5S	Broad range	High sensitivity and sensitive to nitrogen oxides

**Table 7:** The content of aldehydes in pork patties with different replacement proportions of AB stalks (%).

No	Compound	C	P25	P50	P75	P100
1	Octanal	0.552	0.336	0.883	0.337	ND
2	Nonanal	2.188	3.328	4.158	2.423	2.135
3	Decanal	1.318	ND	2.349	0.263	ND
4	Benzaldehyde	2.359	3.829	2.38	1.291	3.045
5	2,4-Decadienal, (E,E)-	1.225	1.959	3.155	6.599	16.614
6	Butanal, 2-methyl	0.586	0.395	ND	0.284	ND
7	Pentanal, 3-methyl-	6.215	8.995	8.731	8.554	ND
8	2-Octenal	ND	ND	ND	0.78	1.147
9	2-UNDecenal	ND	ND	ND	0.34	0.727
10	2-Heptene aldehyde	ND	ND	ND	ND	1.067
11	2-Decenal, (E)-	ND	ND	ND	0.341	1.097
12	4-Methylhexanal	ND	ND	ND	ND	0.477
13	2,4-Decadienal, (E,E)-	ND	ND	ND	ND	1.069
Total		14.443	18.842	21.656	21.212	27.918

ND: not determined. C, P25, P50, P75, and P100 represent 0%, 25%, 50%, 75%, and 100% substitution of pork lean meat by AB, respectively.

**Table 8:** The content of Syringol in pork patties with different replacement proportions of AB stalks (%).

No	Compound	C	P25	P50	P75	P100
1	Methanethiol	0.227	ND	ND	ND	ND
2	1-Octen-3-ol	0.915	0.665	0.855	0.962	0.859
3	1-Hexanol, 2-ethyl-	2.558	1.713	ND	1.188	ND
4	Benzyl alcohol	ND	0.867	1.659	2.903	3.388
5	1-Hexanol	ND	ND	1.016	ND	ND
6	1-Hexanol, 2-ethyl-	ND	ND	1.815	ND	ND
7	2-Furanmethanol	ND	ND	ND	0.211	ND
Total		3.7	3.245	5.345	5.264	4.247

ND: not determined. C, P25, P50, P75, and P100 represent 0%, 25%, 50%, 75%, and 100% substitution of pork lean meat by AB, respectively.

**Table 9:** The content of Terpenes in pork patties with different replacement proportions of AB stalks (%).

No	Compound	C	P25	P50	P75	P100
1	Nonane, 5-methylene-	0.435	ND	ND	ND	ND
2	1-Decene	0.412	0.486	0.335	0.472	0.859
3	Caryophyllene	ND	0.257	0.417	ND	ND
4	5-Tetradecene, (E)-	0.211	ND	ND	ND	3.388
5	2-Nonene, 3-methyl-	0.758	0.921	0.579	0.892	ND
6	4-Nonene, 5-methyl-	ND	ND	0.441	0.377	ND
7	3-Ethyl, 2-methyl-	ND	ND	0.283	0.702	ND
8	2-Dodecene	ND	ND	ND	0.467	ND
9	1-Undecene, 2-methyl-	ND	0.62	ND	ND	ND
Total		1.816	2.284	2.055	2.91	4.247

ND: not determined. C, P25, P50, P75, and P100 represent 0%, 25%, 50%, 75%, and 100% substitution of pork lean meat by AB, respectively.

**Table 10:** The content of Ketones in pork patties with different replacement proportions of AB stalks (%).

No	Compound	C	P25	P50	P75	P100
1	Acetoin	1.06	0.875	ND	ND	ND
2	Acetophenone	0.605	ND	ND	ND	ND
3	5-Chloro-5-methylnonane	ND	0.39	ND	ND	ND
4	5-Hepten-2-one, 6-methyl-	ND	0.304	ND	ND	ND
5	3-Octanone	ND	ND	0.772	ND	ND
6	5,9-UNDecadien-2-one	ND	ND	0.338	ND	ND
7	2-Heptanone	ND	ND	1.468	ND	ND
Total		1.665	1.569	2.578	ND	ND

ND: not determined. C, P25, P50, P75, and P100 represent 0%, 25%, 50%, 75%, and 100% substitution of pork lean meat by AB, respectively.

**Table 11:** The content of Acids in pork patties with different replacement proportions of AB stalks (%).

No	Compound	C	P25	P50	P75	P100
1	Acetic acid	1.413	0.648	1.736	1.413	ND
2	Butanoic acid	0.986	1.013	1.142	0.601	ND
3	Hexanoic acid	0.832	1.292	1.467	0.99	0.488
4	Octanoic acid	0.325	0.372	ND	ND	ND
5	Nonanoic acid	0.575	0.346	0.375	ND	ND
6	n-Decanoic acid	1.043	0.69	0.39	ND	ND
7	Tetradecanoic acid	0.493	0.646	0.493	0.479	0.341
8	Cis-7-Hexadecenoic acid (7Z)	0.341	ND	ND	ND	ND
9	Pentadecanoic acid	0.257	0.255	ND	0.197	ND
Total		6.265	1.569	2.578	ND	ND

ND: not determined. C, P25, P50, P75, and P100 represent 0%, 25%, 50%, 75%, and 100% substitution of pork lean meat by AB, respectively.

**Table 12:** The content of Esters in pork patties with different replacement proportions of AB stalks (%).

No	Compound	C	P25	P50	P75	P100
1	Ethyl Acetate	1.633	1.074	3.503	0.689	ND
2	Acetic acid, butyl ester	1.86	2.283	1.523	ND	ND
3	Propyl acetate	ND	ND	ND	0.254	ND
4	Acetic acid, hexyl ester	0.224	ND	ND	ND	ND
Total		3.717	3.357	5.026	0.943	ND

ND: not determined. C, P25, P50, P75, and P100 represent 0%, 25%, 50%, 75%, and 100% substitution of pork lean meat by AB, respectively.

**Table 13:** The content of Alkanes in pork patties with different replacement proportions of AB stalks (%).

No	Compound	C	P25	P50	P75	P100
1	Octane, 4-ethyl-	0.35	0.248	0.522	ND	0.56
2	2,7-dimethyluNDecanone	0.227	0.323	ND	ND	ND
3	Nonane, 3-methyl-5-propyl-	1.008	0.779	ND	1.058	0.886
4	Undecane, 3-methyl-	1.935	2.911	ND	1.631	3.647
5	Dodecane	2.952	3.765	3.816	ND	ND
6	Tetradecane	0.56	0.498	ND	ND	ND
7	Decane, 4-ethyl-	ND	0.278	0.495	0.285	ND
8	Cyclooctasiloxane, hexadecamethyl-	ND	0.333	0.729	ND	0.503
9	Nonane, 2-methyl-5-propyl-	ND	ND	1.259	ND	ND
10	UNDecane, 3-methylene-	ND	ND	ND	0.333	ND
Total		7.032	9.135	6.281	3.307	5.596

ND: not determined. C, P25, P50, P75, and P100 represent 0%, 25%, 50%, 75%, and 100% substitution of pork lean meat by AB, respectively.

**Table 14:** Other volatile compound content of pork patties with different replacement proportions of AB stalks (%).

No	Compound	C	P25	P50	P75	P100
1	Maltol	0.376	0.366	ND	ND	ND
2	Furan, 2-ethyl-	ND	0.182	0.29	0.383	ND

ND: not determined. C, P25, P50, P75, and P100 represent 0%, 25%, 50%, 75%, and 100% substitution of pork lean meat by AB, respectively.

**Table 15:** Microbiological analysis of pork patties.

Microorganisms (log CFU/g)	C	P25	P50	P75	P100
Total viable counts	1.35 ± 0.30 ^a^	1.65 ± 0.35 ^a^	1.15 ± 0.15 ^a^	0.85 ± 0.15 ^a^	1.55 ± 0.25 ^a^
Coliform	ND	ND	ND	ND	ND
Staphylococcus aureus	ND	ND	ND	ND	ND
Mold	ND	ND	ND	ND	ND

^a^: Statistical differences between different sample groups are shown with different. ND: not determined. C, P25, P50, P75, and P100 represent 0%, 25%, 50%, 75%, and 100% substitution of pork lean meat by AB, respectively.

## Data Availability

The original contributions presented in the study are included in the article, further inquiries can be directed to the corresponding author.
